# Enriched gardens improve cognition and independence of nursing home residents with dementia: a pilot controlled trial

**DOI:** 10.1186/s13195-021-00849-w

**Published:** 2021-06-16

**Authors:** Etienne Bourdon, Joël Belmin

**Affiliations:** 1 Hôpital Charles Foix, Groupe hospitalier APHP.Sorbonne, 7 avenue de la République, 94200 Ivry-sur-Seine, France; 2 Laboratoire Education et Pratiques en Santé (LEPS), EA3412, Université Paris Sorbonne Nord, Bobigny, France; 3 Faculté de Médecine, Sorbonne Université, Paris, France

**Keywords:** Enriched environment, Dementia, Alzheimer’s disease, Enriched garden, Cognitive function, Functional autonomy, Risk of falls, Conventional sensory garden, Nursing homes

## Abstract

**Background:**

Dementia is a major issue worldwide, and considerable efforts were made to design therapeutic mediation tools and evaluate their benefits on the health of patients.

**Methods:**

**Design:** Multi-center cluster-controlled pilot trial.

**Settings and participants:** Four nursing homes that offered separated access to one conventional sensory garden (CSG) and one enriched garden (EG). The participants were residents with dementia, independent for walking and with no severe dementia or behavioural troubles. Eligible residents were divided into three groups according to the proximity of their room: close to the CSG or EG gardens for the first two groups and further from the gardens for the third (control) group.

**Interventions:** We asked staff members to frequently invite residents to visit the EG or the CSG depending on their group allocation. No invitation to gardens was made to the control group. We installed 12 enrichment modules in the EG that stimulated cognitive, independence and walking/balance functions.

**Measures:** Cognitive function (MMSE), independence for activities of daily living (ADL) and risk of falls (unipodal stance and timed up and go – (TUG)) were assessed at baseline and after 6 months.

**Results:**

The 120 participants were 81·0 ± 3·5 years old and comprised of 83 women. Their MMSE score was 17·5 ± 2·9. Patients’ characteristics were not significantly different between the three groups. Among the participants invited to visit the EG group, 6-month changes in MMSE showed improvement compared to other groups (+ 0·93 ± 0·65 vs −0·25 ± 0·71 and −0·24 ± 0·73 in the EG vs CSG and control groups, respectively, *P* < 0·0001). Changes in ADL, TUG and unipodal stance were significantly improved in the group visiting the EG as compared to other groups, which indicates better functioning.

**Conclusions:**

EGs offer a new approach to therapeutic mediation for residents of nursing homes with dementia.

**Supplementary Information:**

The online version contains supplementary material available at 10.1186/s13195-021-00849-w.

## Introduction

Alzheimer’s disease and other dementias are the leading causes of disability, dependence and institutionalization in the elderly. No drug therapy [1] effectively cures this disease, and approaches to improve patient’s quality of life are essential. The well-being of nursing home residents is a major goal of care, which is positively affected by many factors such as appropriate medical care, nutrition, housing, everyday care and good relationships and communication with staff members, family members and other residents [2]. Environmental design may also participate in the well-being of nursing home residents, and the concepts of therapeutic or dementia-friendly environments [3] have emerged from the literature. Recently, the Interdem Social Task Force supported, through an expert consensus, the idea that the design of the physical environment is a promising way to improve the social health of people with dementia [4]. These environments are designed to compensate for impairments related to advancing age and neurocognitive disease.

Environmental enrichment produced favourable brain and cognition effects in experimental animals and also in humans. Donald O. Hebb [5, 6] demonstrated in his pioneering experiments in 1947 that a defined favourable environment for the breeding of rats significantly influenced on their ability to solve problems compared to a population of rats raised in ordinary cages. Further exploratory works [7, 8] showed that an enriched environment increased the cerebral cortex thickness of rats raised in cages and valorized the notion of an enriched environment as a positive factor influencing health. Berardi and Maffei [9] highlighted the positive impact of enriched environment in a mouse model of Alzheimer’s disease in the early 2000s, by delaying the progression of neurodegeneration and preventing the onset of memory deficits. Recently, environmental enrichment was found to induce an increase in brain capillary density and improved cognitive performance in aged mice [10]. The effects of enriched environments have been studied in several animal studies, but very few models have been transposed to humans according to the principles of translational research, with the notable exception of autism. In two randomized controlled trials conducted by the same team, a 6-month exposure to an enriched sensorimotor environment ameliorated autism-like symptoms in autistic children [11, 12].

However, whether an enriched environment would have a positive effect on humans with dementia was not known. Based on the existing literature [13–15], we identified that gardens in nursing homes were valuable candidates for translational research on enriched environment and evaluate the benefits of an enriched environment on residents with Alzheimer’s disease. To answer this question, we designed a pilot cluster trial to determine whether environmental enrichment applied to gardens in nursing homes could produce beneficial effects on clinical markers of function for residents with dementia.

## Methods

### Study design and participating nursing homes

**Fig. 1:**
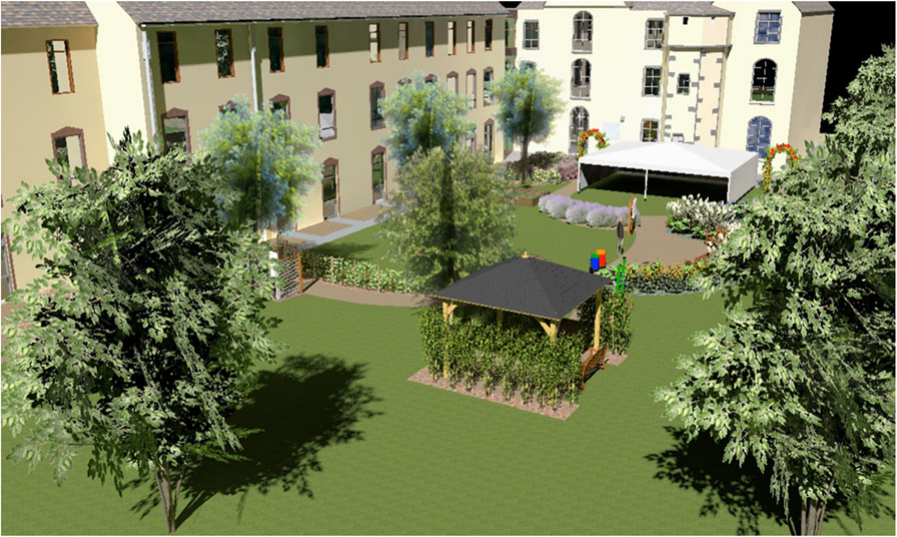
A 3D general overview of the conventional sensory garden (nursing home # 2). The conventional sensory garden at each facility offered beds of perennials, shrubs and trees, with an emphasis on the variety of sensory stimulation, garden furniture sets and ergonomic planters for gardening. The gardens area was 380 m^2^ for facility #1, and 590, 540, 450 for facilities #2, 3 and 4, respectively)

**Fig. 2:**
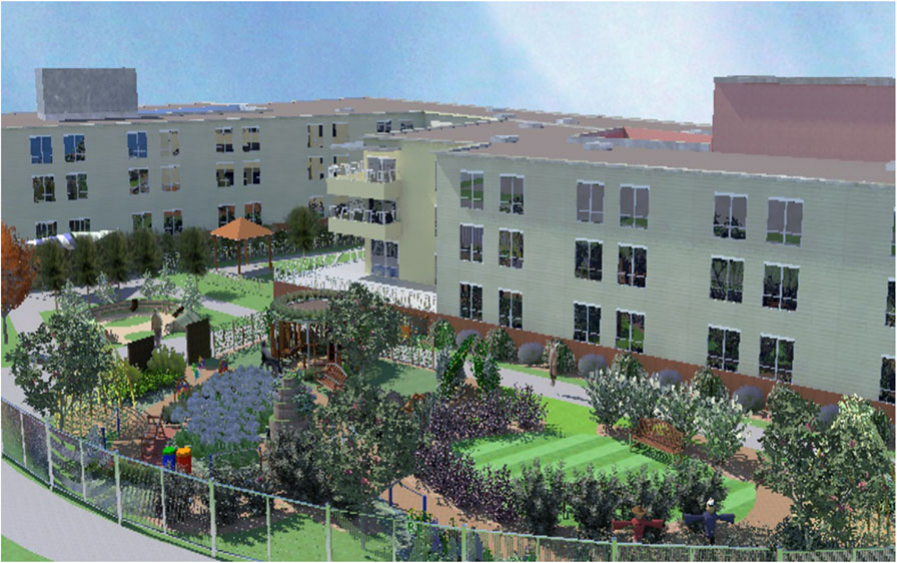
A 3D general overview of the enriched garden (nursing home #2). The enriched garden at each facility included 12 specially designed pieces of equipment that targeted cognitive stimulation, support of autonomy and fall prevention. The different enrichment modules were designed to be integrated into a garden, and it appeared that these items were garden constituents. The gardens area was 530 m^2^ for facility #1, and 450, 350 and 410 for facilities #2, 3 and 4, respectively)

This multi-center cluster-controlled pilot trial was performed in four French nursing homes that had both a conventional sensory garden and an enriched garden that was designed by a specialized landscape architect. These four nursing homes were selected to participate in the study because they all had an enriched garden and a conventional sensory garden, which is not a common situation. We obtained from garden design companies, a list of nursing homes where an enriched garden had been installed in recent years. We contacted them to find out whether they also had a conventional sensory garden and whether the access was separate from the enriched garden. Finally, we asked to the directors of these facilities if they were interested in participating to the study. Overviews of the conventional sensory garden and an enriched garden of the nursing home # 2 are shown in Figs. 1 and 2. The outdoor gardens were separated and had specific access points, with no possibility to directly travel between gardens (supplementary material Figure 1). The gardens were open to residents during the day and closed at night.

**Fig. 3:**
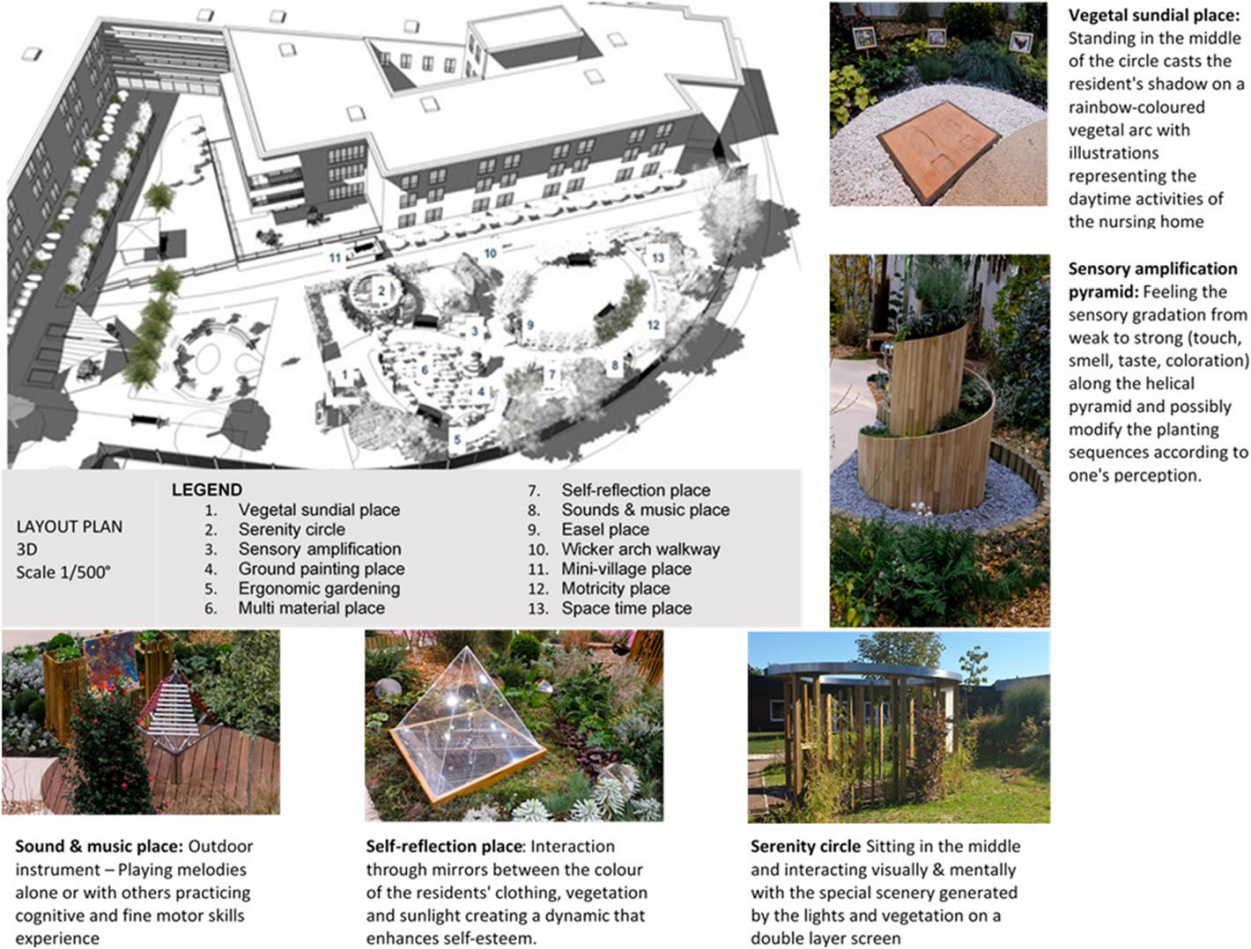
A 3D layout plan of the enriched garden (nursing home #2) showing the distribution of the stimulating modules along an enriched garden, and the description with pictures of 5 of these modules

The garden enrichment resulted from specific research and conception ideas to design the adapted facilities for implementation based on therapeutic goal [16]. Each enriched garden module was conceptualized as a co-construction process with several staff members and architectural teams. Table 1 shows the types of activities or environments implemented in the enriched gardens and the purpose of the designers. Places of activities and particular environments in the enriched garden were designed for intuitive use without any particular written or oral instruction or human facilitation. The enriched garden surface areas were 300 to 600 m^2^. Distribution of the modules in the enriched garden of the nursing home #2 and examples of modules are shown in Fig. 3.

**Table 1:** The stimulation modules implemented in the enriched garden, their purpose and their description with activities

Module	Purpose	Description (D) and activities with approximative time spent in activities (A)
1 - Vegetal sundial place	Cognition impairment Temporo-spatial disorientation	D: On sunny days, the shadow projection of the patient gives him indication of daytime over a half circle multi-colour flowerbed—it is associated with a distribution of the same colors over different milestones throughout the garden pathway A: Standing & measure day time and walking over colored milestones (2–5 min)
2 - Easel place	Cognition impairment Emotional praxis	D: Free expression with a paintbrush over a rain washable canvas of instantaneous emotions that will remain as a garden land art expression. It will exercise motricity of superior members, cognitive stimulation and spatial representation A: Painting with a brush in hand on a washable surface (5 min)
3 -Self-reflection place	Self-esteem Mild depressive disorder	D: Interaction of reflection of the patient with mirrors distributed in a specific spot producing a magnified light and colour effects through pyramidal prisms with vegetal planted beds A: Standing and interacting visually & mentally with the special scenery (5 min)
4 - Space-time place	Cognition impairment Temporo-spatial disorientation	D: Architectural construction catching natural lights and producing special effects in its environment during the day A: Standing or sitting and observing/interacting with the special light effects along the day created by sun rays (5 min)
5 -Sensory amplification	Sensitive stimulation	D: Pyramidal construction of tactile, olfactory and visual effects with a gradated selection of vegetal planting A: Feeling the sensory gradation of this helicoidal pyramid and possibly modify planting (5–10 min)
6 - Wicker arch walkway	Cognition impairment Praxis	D: Wicker arch constructed over the garden pathway with braiding and shadowing calming interaction A: Braiding of wicker strands (3–5 min)
7 - Ground painting place	Cognition impairment Praxis	D: A long vertical paintbrush moving along a cable via gravity produces colored paint traces over a specially designed surface as the patient walks while holding the brush handles. A: Accompanying a cable suspended brush and leaving traces of painting on a special surface (3–5 min)
8 -Motricity place	Walking, exercising balance, prevention of falls	D: Different pathways between parallel bars for exercise on slopes, stairs and obstacles crossing A: Crossing obstacles (2–3 min)
9 - Sounds and music place	Cognition impairment Praxis	D: Different outdoor musical instruments with predesigned or free musical exercises A: Playing alone or with others melodies on outdoor specific music instruments (5 min)
10 -Multi-materials place	Walking, exercising balance, prevention of falls, multisensory interaction	D: Special construction to experience differences in materials by touching, viewing, walking and smelling A: Crossing multi materials path and challenging vestibular sensation (2–3 min)
11 - Ergonomic gardening	Cognition impairment Praxis	D: Flower and vegetable bed planters built with ergonomic access for patients either in wheelchairs or with limited mobility A: Re-visited gardening activities centered on cognitive and functional experience (5–10 min)
12 - Serenity circle	Troublesome & disruptive behaviour	D: Specially designed semi-closed place using light vegetation and vertical wooden bars to offer a safe and harmonious environment that includes a double layer tight canvas producing special effects in interactions with light and shadows A: Sitting in the middle and interacting visually & mentally with the special scenery (7–10 min)

### Participants

We have defined in each nursing home three sectors corresponding to care units. Depending on the location and its proximity to the gardens, one sector was considered close to the enriched garden, another was considered close to the sensory garden and the latter was considered not to be near any garden. Participants were assigned to one of the three groups based on the location of their room in those sectors.

Residents of these sectors were eligible for the study if they had Alzheimer’s disease or another type of dementia and were capable walking independently with no human help. The diagnosis of Alzheimer’s disease or another type of dementia was made by the general practitioner and/or the medical coordinator of the facility and was explicitly mentioned in the resident’s medical record. Patients with a severe cognitive deficit which was defined by an MMSE score<10 and patients with severe behavioural problems were excluded. All eligible residents were asked to participate in the study and were allocated to one of the three groups based on the location of their room in the units: Conventional garden group, enriched garden group or control group.

### Interventions

The interventions were performed for 6 months during the spring and summer. For the participants of the conventional sensory garden and enriched garden groups, the intervention aimed to encourage them to frequently visit the conventional sensory gardens. For participants of the control group, usual care was applied and no specific intervention was done to encourage them to visit gardens.

For the conventional sensory garden group, we asked the staff members of their unit to remind and invite the participants to visit the conventional sensory garden. We asked them to do it several times a day in order to get 4 visits a week per participant. We also asked to staff members to accompany residents to the entrance of the garden and to encourage them to take a walk. Residents usually walked around the garden alone or with other residents during 10–20 min, and we did not ask to staff members to accompany them during the visit to garden. Staff members were also asked to invite families to use the corresponding garden with their relative.

For the enriched garden group, we asked the staff members of their unit to remind and invite the participants to visit the enriched garden with the same recommendations with regarding the frequency of the visits. In addition, soon after participants inclusion, staff members were asked to introduce the enriched garden modules to each resident in this group, during a short individual visit of about 15 minutes. Then, over the next few months, each resident had their own experience of interacting with the modules, which was left to intuition. The path through the garden from one visit to the next could be different, with the resident not necessarily stopping at the same stations each time.

For the control group, we did not give any instruction to staff members about visits to gardens, and therefore, they did not give to these residents specific invitations to visit the gardens.

During the study, free access to the gardens was possible through doors which opened and unlocked automatically from 8 a.m. to 8 p.m, and access to the gardens was not restricted to any person in the facility. So all the residents could visit any garden, whether or not they were participating in the study and regardless of the group to which they were assigned. Of course, visitors and residents’ families were welcome to accompany residents to the gardens.

### Staff member information

Prior to starting the study and implementing the interventions, we organized two 2-h meetings for all the staff members, irrespective of the units they were working in. In each of the 4 nursing homes, 10 to 12 staff members participated in one of these meetings. At these meetings, one of us presented the aim of the study and the role of the staff members in implementing the interventions, which was to encourage participants assigned to the conventional sensory garden or the enriched garden to visit their respective garden (conventional sensory garden and enriched garden) at least 4 times a week and also to ensure that the residents visited their garden regularly. We did not give instructions to staff members about accompanying residents into the garden and spending time with them. With the exception of a few specific activities, most of the residents’ visits took place without professional assistance.

### Measurements

For the participating residents of each facility, two observers (a psychologist and an occupational therapist), who were independent of the research team, performed the following assessments at inclusion and after 6 months: global cognitive function using the Mini-Mental Status Examination (MMSE), the level of independence using activities of daily living (ADL), and gait and balance using the timed up and go test (TUG) and unipodal stance tests respectively. The raters were not provided with a description of the protocol. They therefore assessed each participant without knowing the purpose of the study and the participant allocation group. According to the blind assessment process, we did not measure the time spent by residents in the gardens. Additionally, we are unable to accurately describe the percentage of time residents use the gardens independently. In average, each nursing home had staff led activities in gardens (#45 min) twice a month mostly between May and early September. Thus, most of the time spent by residents in the gardens was done without the presence of staff members.

### Ethics

The ethics committee (Comité de Protection des Personnes IDF VII, France) approved the study, which was performed according to the principles of the Declaration of Helsinki.

### Statistics

The characteristics of the residents were compared using the chi-squared test and one-way ANOVA. Changes in MMSE, ADL, TUG, and unipodal stance were compared using repeated measures ANOVA. Analyses were performed using STATA version 16 (StataCorp LLC, College Station, TX, USA). P<0.05 was considered the level of significance.

## Results

Among the 368 residents of the four facilities, 220 (60%) had the diagnosis of Alzheimer disease or other type dementia, and 266 (72%) were able to walk alone. A total of 140 residents were included in the study after screening eligible participants in the four nursing homes (flowchart suppress in supplementary material Figure 2). Seventeen dropouts occurred during the 6-month follow-up, including 6 in the control group, 5 in the conventional sensory garden group and 6 in the enriched garden group. These dropouts resulted from events that occurred during the trial, such as hospitalization and loss of walking independently. Data were missing for 3 residents, and this data were not included in the final results. Therefore, the data of 120 residents were analysed, 39 residents in the control group, 41 residents in the conventional sensory garden group and 40 residents in the enriched garden group (flowchart in supplementary material Figure 2).

The residents’ characteristics in each group are shown in Table 2. No significant differences were observed between the 3 groups in age, gender, baseline MMSE, ADL, TUG or unipodal stance values.

**Table 2:** Characteristics of the participants

	No incentive to visit gardens (*n* = 39)	Incentive to visit conventional sensory gardens (*n* = 41)	Incentive to visit enriched gardens (*n* = 40)	*P*
Age (years)	81.1 ± 3.5	80.5 ± 3.6	80.9 ± 3.5	0.70
Women	26 (67%)	28 (68%)	29 (72%)	0.81
Setting				
I	12 (31%)	10 (24%)	7 (18%)	0.93
II	8 (21%)	10 (24%)	9 (23%)	
III	13 (33%)	16 (39%)	17 (43%)	
IV	6 (15%)	5 (12%)	7 (18%)	
MMSE score (0–30)	17.3 ± 3.3	17.8 ± 2.9	18.0 ± 2.7	0.57
ADL (0–6)	4.28 ± 0.69	4.29 ± 0.66	4.27 ± 0.54	0.98
3 ADLs	10 (25%)	8 (19%)	6 (15%)	0.37
4 ADLs	17 (44%)	20 (49%)	26 (65%)	
5 ADLs	12 (31%)	13 (32%)	8 (20%)	
Unipodal stance (s)	8.03 ± 4.23	8.85 ± 4.67	8.65 ± 4.60	0.70
< 5 s	10 (26%)	10 (24%)	10 (25%)	0.91
5 to 10 s	17 (43%)	15 (37%)	14 (35%)	
>10 s	12 (31%)	16 (39%)	16 (40%)	
Time up and go (s)	15.69 ± 4.54	14.54 ± 3.81	15.53 ± 5.24	0.47
< 15 s	14 (36%)	22 (54%)	20 (50%)	0.11
15 to 20 s	15 (38%)	16 (39%)	10 (25%)	
>20 s	10 (26%)	3 (7%)	10 (25%)	

*MMSE* Mini-Mental Status Examination, *ADL* activities of daily living

During the 6-month follow-up, we observed a functional decline in the conventional sensory garden and control groups in MMSE, ADL, TUG and unipodal stance values. However, significant and positive effects on MMSE, ADL, TUG and unipodal stance values were observed in the enriched garden participants (Table 3). The percentages of residents with improvements in independence, TUG and unipodal stance values were significantly greater in the enriched garden group compared to the two other groups (Table 3). We did not record any adverse events related to garden use.

**Table 3:** Changes from baseline in the Mini-Mental Status Examination, the independence for activities of daily living (ADL), unipodal stance and timed up and go tests

	No incentive to visit gardens (*n* = 39)	Incentive to visit conventional sensory gardens (*n* = 41)	Incentive to visit enriched gardens (*n* = 40)	*P*
Mini-Mental Status Examination Score	−0.25 ± 0.71	−0.24 ± 0.73	+0.93 ± 0.65	0.0001
Independence for ADL	−0.05 ± 0.32	−0.12 ± 0.24	0.30 ± 0.35	0.0001
Worsen (−1)	5 (13%)	9 (22%)	1 (2%)	<0.0001
No change	30 (77%)	32 (78%)	18 (45%)	
Improved (+1)	4 (10%)	0	21 (53%)	
Unipodal stance (s)	−1.10 ± 2.09	−0.46 ± 3.49	+1.78 ± 3.84	0.0007
Worsen	39 (100%)	15 (37%)	0	<0.0001
No change	0	16 (39%)	0	
Improved	0	10 (24%)	40 (100%)	
Timed up and go (s)	+0.77 ± 2.71	+0.51 ± 3.17	−1.95 ± 2.98	0.0001
Worsen	15 (38%)	18 (44%)	5 (12%)	0.001
No change	17 (44%)	15 (37%)	14 (35%)	
Improved	7 (18%)	8 (19%)	21 (53%)	

## Discussion

This pilot study showed that incentives for nursing home residents with dementia to attend an enriched garden contributed to better functioning compared to residents who were invited to visit a conventional sensory garden or who were not invited to visit a garden. The concept of enrichment environments placed in gardens for Alzheimer’s disease patients is a new approach to improve the functioning of demented patients.

Previous studies highlighted the beneficial contribution of gardens to Alzheimer’s disease patients [17–19]. Whear et al. [20] published a systematic review in 2014 to assess the effects of gardens on the health of nursing home residents with dementia. They identified ten quantitative studies, all with methodological limitations and a high risk of bias. Most studies investigated dementia-related behaviour, and 6 studies revealed a favourable effect on agitation. The gardens in these studies were not specially designed for nursing home residents, except in the study by Edwards [21] in which the garden that was specially designed for nursing home residents with dementia. In their scoping review, Howarth [17] et al. identified 14 studies which assessed the effects of gardening on patients with dementia and they concluded that it contributes to positive behaviour changes and better quality of life.

Our study on the effects of enriched gardens on nursing home residents with Alzheimer’s disease is original and innovative in several aspects. The design of the enriched gardens was inspired by the conceptual model of Hebb [5, 6] and followers [7–10, 22–24] based on enriched environment in which the implementation of stimulating devices in the environment had positive effects on a variety of brain functions in both animal and human studies. We applied this concept to the context of nursing homes and designed enriched gardens comprising a variety of stimulating modules, as described in Table 1. As the beneficial effect of walking outside and visiting outdoor gardens is well documented among nursing home residents [19], we have conceptualized an enriched environment within a garden. To explore the specific effects of this enriched environment, we conducted this study in facilities that had both conventional sensory gardens and enriched gardens, to compare the effects of the two types of gardens in the same facilities. Both types of gardens offered similar interactions with nature, including an open-air walking path and an atmosphere of well-being, but only the enriched gardens comprised the specific modules designed to address dementia related troubles. The availability of the two types of gardens in the same facility is an infrequent occasion and also a remarkable point of our study, that led us to the conclusion that the enriched garden offered specific beneficial effects compared to conventional gardens.

In our study, the better effects observed in the enriched garden group suggest that the stimulating modules are the main active component acting in combination with favourable effects provided by the garden. We based our hypothesis on the idea that the open-air and vegetal atmosphere of the landscape garden favourise letting go by the visiting resident and create favorable conditions to interact with the existing stimulating modules. Each of twelve modules was designed to focus on specific weaknesses or disorders of residents with Alzheimer disease. In this multimodal approach, several modules were conceptualized to stimulate cognitive abilities, walking abilities and independence. Following the intervention, we observed changes in the corresponding outcomes during the trial, which were greater for residents assigned to enriched gardens than for those assigned to conventional sensory gardens. Although we did not track the effects of each individual module, we designed the intervention with 12 different modules that individually address the specific weaknesses of residents with dementia. Seven of them were designed to stimulate cognitive impairment and eight to stimulate the ability to walk and independence. It is therefore plausible that the interaction with the modules in the enriched gardens had beneficial effects on the outcomes we measured, although our study cannot ascertain this point. A better demonstration could be obtained by future studies recording in detail the interaction of residents with specific modules and examining the relationship between these interactions and the clinical effects, but this goal was far beyond the scope of this pilot study. We observed in the participants of the group enriched garden a significant improvement in cognition that exceeded our expectations. Although these effects on cognitive and physical function were small, they were statistically significant and this finding is very promising in the face of a disease for which many treatments have been shown to be ineffective. This is consistent with a body of literature showing that cognitive stimulation can have positive effects on cognition of residents with Alzheimer’s disease [25]. Interestingly, in another context, Then et al. showed that an enriched environment at work place was protective from incident dementia (odds ratio 0.61, 95%CI: 0.47–0.79) in the Leipzig longitudinal study of the aged [26, 27], and the authors explained their finding by the effect of enriched environment on cognitive abilities and cognitive reserve.

### Limitations

Our study has several limitations. Our pilot study was not a randomised trial, and patients were assigned to groups based on the location of their rooms in relation to the gardens. This pragmatic design made it possible to compare the different groups, and it would have been technically and ethically difficult to set up a trial with a randomization of residents. Fortunately, we did not observe large baseline differences between the three groups, but we cannot exclude a possible selection bias. We also did not record the attendance of the participants in the gardens nor the duration of their use, and for the enriched garden group, the number of interactions with the stimulation modules. We did not specifically measure family or professional caregiver participation in garden visits with residents, which could have had positive effects. From our clinical experience, if it was clear that staff members of the facilities felt interested by the availability of gardens, they did not spend a lot of time in gardens with residents due to their heavy workload. In addition, we did not measure staff attention for the residents which might have been different between groups and might also represent a potential bias. Finally, in our pilot study, we did not assess the effects of enriched gardens on behavioural and psychological symptoms which are an important issue for certain residents with dementia, nor on social inclusion, self-esteem or perceived well-being (Howarth, 2017) [17].

## Conclusions and implications

Our study suggests that enriched gardens represent a new approach to therapeutic mediation for residents of retirement homes with dementia via the offering of stimulating psychomotor activities performed in an open-air garden setting. The results of our pilot study must be confirmed in a large-scale trial that includes a detailed monitoring of garden use by residents and caregivers, and that of residents’ behavioural symptoms and quality of life. The application of the enriched environment concept to nursing homes is a promising approach to improve the cognition, independence and daily lives of residents and alleviate the insufficiently stimulating atmosphere of many facilities.

## Supplementary Information

**Additional file 1.**

## Data Availability

The datasets used and/or analysed during the current study are available from the corresponding author on reasonable request.
