# Supplementary material for: Enriched gardens improve cognition and independence of nursing home residents with dementia: a pilot controlled trial
Source: Alzheimers Res Ther. 2021 Jun 16;13:116. doi: 10.1186/s13195-021-00849-w (PMC8207740; doi:10.1186/s13195-021-00849-w)
Supplement: Supplementary file 1 — Additional file 1. [file 13195_2021_849_MOESM1_ESM.docx]

**Supplementary material. Figure 1: Layout plan of the conventional sensory garden (left panel) and the enriched garden (right panel) of the nursing home # 1 showing circulation pathways (in brown). Please note that accesses (red arrows) to the two gardens are separate and that there is no direct communication between the two gardens.**


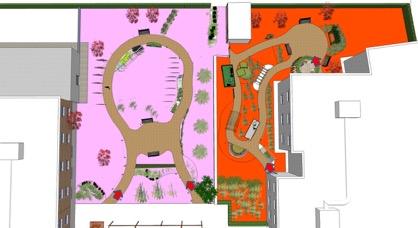


**Supplementary material. Figure 2: Flow chart of participants selection**

Residents in four nursing homes

(n=368)

Residents with

inclusion criteria

(n=140)

* NH : nursing home

245 excluded for:

- No dementia (n=148)

- Severe dementia (n=62)

- Severe behavioural disorder (n=72)

- Inability walking alone (n=102)

Allocation according to the accessibility from their room to

Control group

(n=46)

Conventional sensory garden group

(n=47)

Enriched garden group

(n=47)

Dropped out (n=6)

Missing data (n=1)

Dropped out (n=5)

Missing data (n=1)

Dropped out (n=6)

Missing data (n=1)

Residents analysed

(n=39)

NH1=12

NH2=8

NH3=13

NH4=6

Residents analysed

(n=41)

NH1=10

NH2=10

NH3=16

NH4=5

Residents analysed

(n=40)

NH1=7

NH2=9

NH3=17

NH4=7

No easy access to gardens

Easy access to

conventional garden

Easy access to

enriched garden
